# AI and Microfluidics: Unlocking Cellular Motility for Bioengineering

**DOI:** 10.3390/bioengineering13020172

**Published:** 2026-01-31

**Authors:** Xueying Zhao, Beibei Gao

**Affiliations:** Department of Chemical Engineering, University of Virginia, Charlottesville, VA 22903, USA; bg6hp@virginia.edu

**Keywords:** microfluidics, chemotaxis, cellular motility, artificial intelligence, deep learning, image segmentation, pollutant detection

## Abstract

Cell movement is central to processes in biology, medicine, and environmental science. Microfluidic technologies have opened new possibilities for studying chemotaxis and motility by creating precise chemical gradients and realistic microenvironments, while allowing direct, real-time imaging under a microscope. At the same time, artificial intelligence (AI) has reshaped how we analyze these behaviors, enabling automated image segmentation, cell tracking, and predictive modeling at scales that were previously impractical. Together, AI and microfluidics form a powerful combination. They offer high-throughput, quantitative insights into single-cell and collective dynamics and drive innovations in areas such as pollutant detection and bioremediation. This review explores recent progress in microfluidic design, AI-based analysis, and portable sensing platforms, and discusses the challenges of data standardization, interpretability, and field deployment. These advances point toward a future where intelligent microsystems play a key role in bioengineering and environmental monitoring.

## 1. Introduction

Chemotaxis, the directed movement of cells in response to a chemical gradient, plays a central role in microbial survival, immune responses, wound healing, and environmental biodegradation [1,2]. Traditional assays such as capillary migration or agar plate diffusion lack the spatial stability and resolution required to capture rapid cellular decision-making. Microfluidics has addressed these limitations by enabling stable gradient formation, precise microenvironment control, and high-resolution time-lapse imaging [3,4,5,6].

Recent work has demonstrated the power of microfluidic systems for exploring complex chemotactic behaviors, including responses to competing stimuli and heterogeneous environments [7,8,9]. For example, Zhao and colleagues quantified *Escherichia coli* chemotaxis toward competing attractants in controlled gradients [7] and developed mathematical models to predict these behaviors [8]. Environmental applications include marine bacteria chemotaxis toward crude oil components with opposing effects [9], highlighting the ecological relevance of chemotaxis in pollutant degradation. The role of bacterial chemotaxis in improving bioremediation efficiency has also been emphasized in engineering contexts [10].

Beyond bacterial systems, microfluidic platforms have evolved to support high-throughput single-cell capture and immune profiling using microwell-based technologies [11,12]. These innovations demonstrate the versatility of microfluidics for both environmental and biomedical applications. However, modern microfluidic assays generate large imaging datasets that are impractical to analyze manually. Artificial intelligence (AI) has simultaneously transformed the scale and depth of quantitative motility analysis through automated segmentation, tracking, and predictive modeling [13,14,15]. Deep learning methods such as U-Net, Mask R-CNN, and YOLO have therefore become essential for extracting trajectories, morphological features, and chemotactic indices [16,17]. AI-driven tracking and behavioral modeling now allow researchers to interrogate cellular movement with unprecedented precision and throughput. Beyond analysis, machine learning approaches for defect detection in microwell-based medical devices illustrate the broader integration of AI into microfluidic technology [18]. These advances collectively underscore the convergence of microfluidics and AI as a transformative paradigm for studying chemotaxis and enabling environmental biosensing.

In this review, we present the integration of microfluidics and AI with a focus on applications in cellular motility and chemotaxis, particularly for environmental and biosensing contexts. We first discuss advances in microfluidic technologies for creating controlled microenvironments (Section 2), followed by a review of recent developments in AI-based methods for cell motility and chemotactic behavior (Section 3). Building on these foundations, we highlight emerging examples that combined microfluidics and AI into integrated pipelines for environmental contaminant analysis (Section 4). Finally, we outline current challenges and future perspectives for leveraging AI-enabled microfluidic platforms to advance quantitative, high-throughput analysis for bioremediation (Section 5).

## 2. Microfluidic Technologies

**Figure 1:**
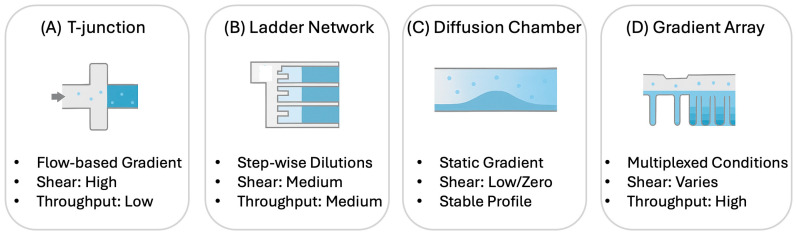
Comparison of major microfluidic chemotaxis platforms, including T-junction, ladder networks, diffusion-only chambers, and multiplexed gradient arrays.

Microfluidic gradient generators, such as T-junctions, ladder networks, and diffusion-only chambers, enable spatially and temporally stable concentration gradients for chemotaxis assays [19]. An illustration for some microfluidic gradient generators is shown in Figure 1. Microfluidic gradient generators also differ in how strictly they control shear stress, temporal stability, and spatial uniformity, which are factors that directly influence cellular behavior and thus the downstream AI workload. Flow-mixing designs such as T-junctions and Y-junctions rely on laminar co-flow and diffusive mixing, producing rapidly adjustable gradients but introducing non-negligible shear stresses along the channel walls. This shear can modulate chemotactic sensitivity and may require AI models to disentangle flow-driven drift from true biased migration. In contrast, ladder networks and serial dilution devices generate quantized, step-wise gradients through controlled hydrodynamic resistance, enabling highly reproducible dose–response profiling ideal for machine-learning-based phenotypic screening. Diffusion-only chambers, particularly those fabricated with hydrogels or porous matrices, recreate near-physiological microenvironments with minimal flow perturbations. These designs provide precise control over chemical landscapes, allowing researchers to study directional migration under physiologically relevant conditions. High-throughput microfluidic platforms have been developed to conduct parallel chemotaxis screens across dozens of gradient conditions, significantly improving reproducibility and experimental throughput [20].

Microfluidics also provides microscale habitat realism, recreating environmental structures such as porous soil matrices, pollutant plumes, and aquatic microenvironments [21,22]. These systems are increasingly used to study microbial responses to hydrocarbons, nitrates, heavy metals, and emerging contaminants under controlled but ecologically meaningful conditions [23]. Such insights are critical for predicting pollutant dispersion and bioremediation efficiency in natural environments.

### 2.1. Gradient Design, Stability, and Calibration

Microfluidic gradient generators can produce linear or complex profiles using controlled mixing networks, ladder resistive circuits, or diffusion-only designs. Practical recommendations include matching hydraulic resistances, mitigating bubble formation, and validating spatial profiles with fluorescent tracers or electrochemical probes. Classic designs from enable arbitrary gradient shapes via networked mixers, while ladder networks generate stepwise dilutions with high reproducibility.

Calibration strategies involve fluorescent tracer mapping (line scans and kymographs), finite-element modeling to validate predicted profiles, and on-chip sensors for real-time concentration monitoring. Advanced systems generate complex stimuli, such as oscillatory or cliff gradients, allowing researchers to probe adaptation, memory, and hysteresis in motility decisions.

### 2.2. Multiplexed and High-Throughput Platforms

Beyond single-gradient devices, multiplexed microfluidic systems have transformed chemotaxis research by enabling simultaneous screening of multiple conditions. Platforms such as gradient-array chips and OrganoPlate^®^-based systems allow parallel assays across dozens of chemoeffector concentrations, drastically improving throughput and reproducibility [24]. These designs integrate automated imaging and AI-driven analysis, reducing manual intervention and accelerating data acquisition.

High-throughput platforms are particularly valuable for environmental microbiology, where diverse pollutants (e.g., hydrocarbons, heavy metals, perfluoroalkyl and polyfluoroalkyl substances) must be tested under ecologically relevant conditions [25,26]. Similarly, drug discovery and toxicity screening leverage multiplexed chips to evaluate compound effects on motility and viability in real time [27]. Coupling these systems with machine learning models enables predictive analytics, linking motility signatures to toxicity or bioremediation potential.

## 3. Artificial Intelligence for Cell Motility

Deep learning (DL) has transformed the analysis of microfluidic chemotaxis experiments by enabling robust segmentation, tracking, and predictive modeling. Convolutional neural networks (CNNs) such as U-Net [28] and generalist models like Cellpose [29] provide accurate cell boundary detection across diverse imaging modalities without extensive retraining. Instance segmentation frameworks such as Mask R-CNN [30] and object detection architectures like YOLO [31] support real-time identification of migrating versus stationary cells, even under cluttered conditions.

**Figure 2:**
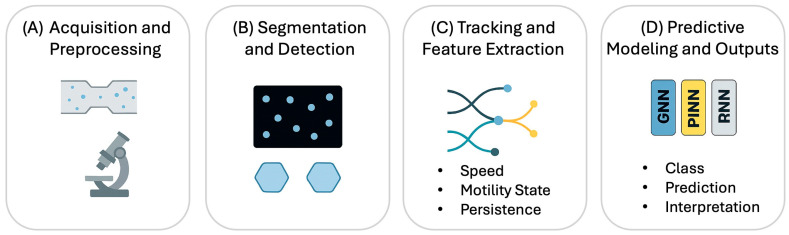
AI pipeline for microfluidic motility analysis, showing acquisition, segmentation and detection, division-aware tracking with feature extraction, and predictive modeling.

For tracking, platforms like TrackMate integrate state-of-the-art segmentation algorithms with advanced linking strategies (Kalman-based, gap closing), supporting batch analysis and reproducible pipelines [32]. Benchmarking initiatives such as the Cell Tracking Challenge have standardized performance metrics (Dice, IoU, AP, mAP, MOTA) and fostered reproducibility across datasets [17]. Dice (Dice Similarity Coefficient) and IoU (Intersection over Union) evaluate segmentation performance by comparing the overlap between predicted cell boundaries and the reference ground truth. AP (Average Precision) and mAP (mean Average Precision) evaluate object detection performance across different confidence thresholds and classes. MOTA (Multiple Object Tracking Accuracy) gauges tracking quality by considering errors such as false positives, missed detections, and identity switches. More recently, HOTA (Higher Order Tracking Accuracy) has emerged as a unified metric that balances detection and association accuracy, providing a fairer assessment of long-term tracking fidelity. Together, these standardized metrics are essential for reproducibility and fair comparisons across algorithms, forming the foundation for community benchmarking efforts. Figure 2 summarizes the AI-based image analysis, while Table 1 lists representative examples of AI applications in microfluidics.

Historically, AI-driven motility analysis relied on descriptive metrics such as Dice, IoU, AP, and MOTA to evaluate segmentation, detection, and tracking performance. These metrics provide a snapshot of algorithm accuracy but do not capture dynamic behaviors or forecast future states. Recent advances move beyond these static descriptors toward predictive modeling of motility dynamics. Graph neural networks (GNNs) learn latent rules linking spatial organization to collective behaviors such as swarming and band formation [37]. Sequence models (Recurrent Neural Network, transformers) capture temporal dependencies in trajectories, forecasting reversals and speed fluctuations. Physics-informed neural networks embed biophysical constraints (e.g., speed limits, gradient bounds) to improve generalization and interpretability.

### 3.1. Analytical Workflows and Behavioral Feature Extraction

Current analysis pipelines ingest raw time-lapse videos acquired in microchannels and proceed through a structured workflow: (i) denoising and normalization; (ii) segmentation and detection to delineate cells; (iii) tracking to conserve identities through time, notably during cell division; (iv) feature extraction of kinematics (speed, persistence, curvature) and morphology; and (v) downstream modeling for interpretation, such as toxicity classification or response forecasting. This workflow culminates in evaluation using the standard metrics (Dice, IoU, MOTA) alongside newer metrics like HOTA, which balances detection precision with association accuracy. Platforms like TrackMate 7 have operationalized these pipelines for biologists by integrating state-of-the-art detectors with configurable linking algorithms (Kalman-based, gap closing) in both 2D and 3D [32].

Microfluidic environments present distinct challenges: low signal-to-noise ratios (SNR), tight channel confinement, and frequent occlusions. In assays involving bacteria or immune cells, rapid divisions often drive identity switches and fragmented tracks. Recent developments address this via Transformer-based linkers, which learn long-range associations and explicitly model division events, thereby reducing reliance on brittle, hand-tuned optimization. Simultaneously, generalist segmenters like Cellpose 2.0 ease the burden of domain adaptation to unique channel geometries, often requiring human-in-the-loop fine-tuning with only ~100–500 ROIs. Together, these advances reduce the gap between descriptive motility summaries and mechanism-aware modeling [36].

A critical nuance in microfluidic analysis is the integration of spatial and temporal information to infer biologically meaningful behavior. In many chemotaxis assays, cells exhibit stochastic movement rather than smooth trajectories. Classical tracking frameworks often penalize these abrupt motions as “tracking errors.” In contrast, modern deep learning models can distinguish biological reversals, pauses, and reorientation events from noise through temporal context modeling. For example, Recurrent Neural Networks (RNNs) and Temporal Convolutional Networks (TCNs) can encode short-term memory of a cell’s directionality, reducing identity switches during transient occlusions. Additionally, attention-based models leverage global relational cues, such as relative cell spacing or boundary proximity, to refine predictions even when local image quality is degraded.

Equally important is the extraction of latent behavioral descriptors beyond traditional kinematic metrics. Deep autoencoders and contrastive learning approaches can map cell morphodynamics (such as subtle shape fluctuations, nucleus–cytoplasm ratios, or polarization signature) into compact latent embeddings. These embeddings have proven powerful for differentiating phenotypically similar subpopulations, such as cells undergoing early versus late chemotactic adaptation, or for identifying rare hypermotile phenotypes that contribute disproportionately to population-level gradients. Such high-dimensional representations provide a fundamentally richer view of motility and chemotaxis than classical centroid tracking, enabling new biological hypotheses to be generated directly from data-driven features.

### 3.2. Comparative Perspective: Architecture Selection for Microfluidic Tasks

Selecting the appropriate AI architecture requires balancing computational cost with the specific biological and physical constraints of the microfluidic environment. For fundamental segmentation, U-Net remains the canonical baseline due to its architectural simplicity and flexibility. However, for new microfluidic datasets, generalist models such as Cellpose and Cellpose 2.0 often provide the most efficient path to robust masking, minimizing the need for extensive retraining. In dense culture environments where precise instance boundaries are critical for maintaining trajectory fidelity, architectures like Mask R-CNN, DeepLab, and StarDist offer superior performance over semantic approaches. Conversely, for portable or edge-based deployments, one-stage detectors, such as the YOLO family, provide a practical balance, delivering sufficient accuracy for on-device screening without the heavy computational overhead of full segmentation.

Tracking algorithms face unique challenges in microfluidics, particularly regarding cell division (mitosis) and occlusion. While classical linkers in TrackMate (utilizing Kalman filters and gap closing) remain reliable for standard assays, the state-of-the-art is shifting toward Transformer-based models. Trackastra has emerged as a powerful solution, directly learning pairwise associations over a temporal window to handle complex lineage branching. This capability resulted in top-tier performance for the Cell Tracking Challenge linking benchmark [35]. Cell-TRACTR extends this paradigm further by performing end-to-end Transformer-based segmentation and tracking, utilizing an adapted Cell-HOTA metric that explicitly scores the fidelity of division events, thereby improving generalization across diverse bacterial and mammalian datasets.

Beyond descriptive trajectory summaries, the field is increasingly adopting predictive architectures. Graph Neural Networks (GNNs) are particularly well-suited for inferring collective migration rules from static spatial snapshots, a direction highly compatible with studies on microfluidic swarming and band formation. To bridge the gap between data and physical reality, Physics-Informed Neural Networks (PINNs) inject governing transport constraints (such as advection-diffusion and Navier–Stokes equations) directly into the training loss. This approach enhances both the plausibility and interpretability of gradient-following predictions within channel flows.

Finally, transfer learning has proven to be a critical enabler for microfluidic imaging tasks where annotated data is limited. A notable example involves Mask R-CNN, which, when fine-tuned with as few as 40 labeled images, accurately quantified droplet and bubble breakup regimes in microchannels. This success demonstrates that pre-trained models can rapidly determine size laws and physical regimes, serving as an instructive paradigm for handling data scarcity in cellular segmentation tasks [38].

### 3.3. Seminal and Recent Case Studies in Microfluidic AI

Recent literature highlights how deep learning is moving beyond proof-of-concept to solve specific biological and engineering bottlenecks. The following case studies illustrate the successful integration of AI with microfluidic hardware to achieve novel functionality.

#### 3.3.1. Robust Lineage Reconstruction in Dense Populations

Bacterial assays present a fundamental tracking challenge: rapid cell proliferation combined with stochastic motility. These factors frequently cause classical tracking software to lose cell identity, resulting in fragmented trajectories that make long-term analysis impossible.

**Figure 3:**
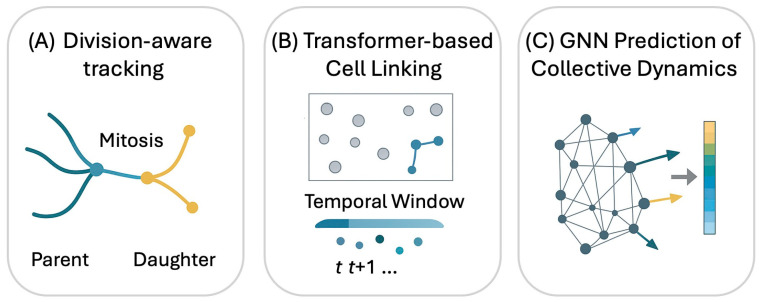
Examples of division-aware cell tracking, transformer-based linking, and GNN-based prediction of collective migration dynamics.

Recent advancements in division-aware tracking address this by maintaining identity across proliferation events (Figure 3A). Trackastra, for example, performs Transformer-based cell linking (Figure 3B) by learning biologically consistent parent–daughter relationships over temporal windows, matching or exceeding the performance of tuned state-of-the-art methods while reducing the need for manual parameter tuning. Similarly, Cell-TRACTR provides a fully end-to-end alternative, introducing metrics like Cell-HOTA to explicitly evaluate lineage consistency. This capability ensures that family trees are reconstructed accurately even in crowded channels, preserving the long-term trajectory identity required for downstream analysis.

#### 3.3.2. AI-Driven Phenotyping of Chemotactic Behavior

Once reliable trajectories are secured, AI models enable a deeper level of behavioral quantification. Chemotactic responses are often characterized by subtle variations in tumbling frequency or gradient-sensing latency. These features are difficult to quantify manually but become readily extractable from unbroken, AI-generated trajectories.

This approach is particularly powerful for revealing heterogeneity. For example, algorithmic analysis can uncover sub-populations with distinct navigational strategies that would be obscured in bulk averages. By coupling microfluidic gradient assays with these high-dimensional behavioral signatures, researchers can detect complex phenotypes such as transient directional coordination or specific run-length distributions that serve as sensitive indicators of environmental sensing.

#### 3.3.3. Label-Free Mechanophenotyping and High-Throughput Screening

A major advantage of combining computer vision with microfluidics is the ability to perform label-free analysis at single-cell resolution. This is particularly valuable in toxicity screens where fluorescent labeling might perturb sensitive cell behaviors [39,40]. Deep learning models can now extract phenotype and motility data directly from intrinsic features in brightfield or phase-contrast microscope images.

In mammalian systems, physical confinement in narrow channels induces specific behaviors such as contact guidance, nuclear deformation, and cytoskeletal reorganization. Deep learning models trained on label-free images are increasingly capable of classifying these mechanophenotypes and correlating them with migration speed or persistence. At the platform level, the integration of robotics with these AI pipelines is reshaping single-cell microfluidics. Automated systems now utilize generative models for batch-effect correction and reducing operator bias and enhancing reproducibility [41].

#### 3.3.4. Structured Micro-Environments for Heterogeneity and Collective Dynamics

New chip formats are being designed specifically to simplify the downstream AI workload. For example, nanowell-in-microwell architectures constrain individual cells, eliminating occlusion and simplifying segmentation. This platform produces clean, labeled datasets directly suitable for training classifiers of motility states (e.g., migrating vs. idle) [42]. When applied to dense populations, these structured environments allow AI to reveal hidden collective behaviors. GNNs can map local neighborhood interactions to emergent dynamics (Figure 3C), enabling the short-term forecasting of phenomena such as band formation or directional coordination, which are events that are driven by the crowd but difficult to predict using traditional statistical averages.

## 4. Environmental Monitoring and Biosensing

Microbial chemotaxis is emerging as a sensitive and biologically relevant approach for detecting environmental pollutants. Unlike conventional chemical sensors, motility-based responses provide real-time insight into how microbes perceive and react to contaminants. Microfluidic platforms that present pollutants, such as Perfluoroalkyl and Polyfluoroalkyl Substances (PFAS), hydrocarbons, pesticides, and heavy metals, enable controlled gradient formation and observation of microbial behavior under ecologically meaningful conditions [43,44,45,46]. These systems not only mimic natural environments but also allow high-throughput screening of multiple compounds in parallel.

**Figure 4:**
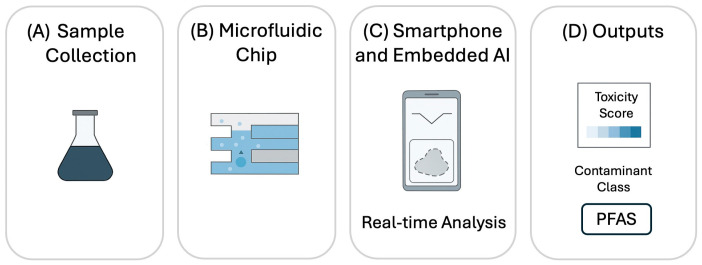
Portable environmental biosensing pipeline integrating microfluidics, smartphone imaging, and edge-deployed AI for toxicity and contaminant detection.

Artificial intelligence adds another layer of capability by analyzing complex motility patterns and predicting toxicity levels. Machine learning models trained on time-lapse imaging can classify chemotactic responses, detect stress-induced changes, and even infer pollutant identity from subtle behavioral fingerprints [15,47]. Wu et al. [15] recently demonstrated this integration by pairing a microfluidic chemotaxis platform with a deep-learning model for automated image analysis. Their method enables fast and precise quantification of cell migration under controlled chemical gradients, significantly reducing manual effort while improving accuracy and reproducibility compared to traditional assays. This work illustrates how combining microfluidics with AI can accelerate applications in environmental biosensing and drug discovery. Figure 4 illustrates this pipeline, showing how field samples are processed into quantitative toxicity outputs.

### 4.1. Practical Deployment Considerations

Deploying AI-enabled microfluidic assays outside of controlled laboratory settings introduces additional engineering constraints. Variability in ambient lighting, temperature, battery life, and sensor noise all affect image quality and must be accounted for through robust preprocessing pipelines, adaptive exposure control, or embedded calibration routines. Smartphone-based microscopy, while accessible, exhibits optical distortions and rolling-shutter artifacts that can complicate segmentation and tracking. These issues can be mitigated by lightweight on-device correction models or by incorporating reference markers within the microfluidic cartridge. Furthermore, real-world environmental samples often contain particulates, autofluorescent debris, or heterogeneous microbial populations. Consequently, classifiers and tracking algorithms must be trained to handle visual ambiguity. Integrating quality-control modules that automatically flag low-confidence outputs can help ensure reliability when assays are operated by non-experts.

Beyond traditional chemotaxis-based toxicity assays, microfluidic platforms are increasingly being engineered to measure a broader suite of environmentally relevant biological responses. For instance, droplet-based microfluidics enables encapsulation of microbial communities or biosensor strains in isolated picoliter compartments, allowing researchers to quantify metabolic inhibition, membrane damage, or oxidative stress in response to pollutants. When paired with AI-powered classification of droplet fluorescence patterns or morphological signatures, these systems can provide rapid estimates of pollutant concentration, toxicity class, or bioremediation potential. AI models can further disentangle the contributions of multiple co-occurring stressors (such as PFAS, nitrates, and heavy metals) by learning multi-modal feature embeddings directly from image sequences.

### 4.2. Portable Imaging and Smartphone-Integrated Platforms

Smartphone-based biosensing and microscopy have advanced to the point where submicron resolution and fluorescence imaging are possible outside the lab [48,49,50,51]. Recent innovations, such as capillary-driven flow devices and compact fluorescence modules (e.g., Pocket MUSE), deliver high-resolution imaging at low cost [51]. These platforms leverage cloud or on-device inference for real-time analysis, reducing latency and expanding accessibility in resource-limited settings. They combine affordability with advanced analytics, making it possible to monitor industrial wastewater or agricultural runoff on-site without relying on centralized labs [52].

For field-deployable applications, reliance on heavy cloud computing is often impractical. A recent breakthrough involves AI-CMCA, a framework designed for capillary-driven chips that operate without external pumps. Using lightweight variants of U-Net and MobileNet-V2, AI-CMCA automates fluid-front detection and path quantification directly on edge devices. It achieves an IoU > 0.9 and analysis speeds 10–100× faster than manual tracking. This case study demonstrates the feasibility of “smart” disposable diagnostics, where the microfluidic chip and the smartphone-based AI analyzer form a self-contained system for reading out motility or gradient propagation.

### 4.3. PFAS and Emerging Contaminants: Sensing Strategies

PFAS remains a major challenge because of their chemical stability and widespread presence in water systems. Microfluidic sensors for PFAS increasingly use interfacial dynamics (such as Janus droplets), aptamer-based recognition, and machine learning to classify species and estimate concentrations in minutes. These approaches tackle issues like specificity, matrix interference, and calibration in complex samples. Researchers are also exploring hybrid sensing strategies that combine optical, electrochemical, and motility-based readouts to improve robustness [45]. Moving forward, standardization and scalable manufacturing will be key to bringing these technologies from the lab to real-world deployment.

## 5. Challenges and Future Perspectives

Despite the substantial progress in AI-enabled microfluidic analysis, the field faces significant methodological and translational hurdles. These challenges span data heterogeneity, model opacity, and the engineering required for robust field deployment. Addressing these limitations is a prerequisite for the transition of these platforms from proof-of-concept to reliable tools in clinical and environmental settings.

### 5.1. Data Variability, Benchmarking, and Standardization

A primary obstacle in applying AI to microfluidics is the lack of uniformity and the limited volume of labeled training data. Unlike natural image benchmarks, microfluidic experiments rarely produce millions of frames with standardized formats. Instead, datasets tend to be small, heterogeneous, and strongly biased toward specific channel geometries or cell types. Substantial variance in image contrast, illumination, and flow rates can introduce domain shifts that degrade model performance. While generalist models like Cellpose 2.0 reduce annotation burdens, they still struggle with the specific artifacts of chemotaxis assays, such as gradient instability or shear-induced morphological changes.

To bridge this gap, researchers are increasingly turning to advanced training strategies. Semi-supervised learning, weak supervision, and synthetic data generation through physics-aware simulation offer promising solutions. For instance, domain-randomized microfluidic simulators can generate physically realistic trajectories and appearance variations, improving model robustness when ground-truth labels are sparse. Similarly, active learning techniques allow models to identify the most informative frames for manual annotation, drastically reducing human effort.

However, generating data addresses only one aspect of the challenge; ensuring consistent reporting is equally critical. One of the most pressing challenges in chemotaxis research is the lack of standardized descriptors for motility metrics such as velocity, persistence, chemotactic index, and turning frequency. This inconsistency makes cross-study comparisons and meta-analyses difficult. Establishing community-driven standards for data formats—including raw images, trajectory files, and metadata—along with reporting checklists will improve reproducibility and enable FAIR (Findable, Accessible, Interoperable, Reusable) data practices [53]. Future efforts must focus on creating microfluidics-specific benchmark datasets to ensure algorithms are evaluated on their ability to handle droplet-interfaces and capillary-driven flows.

Interpretability is equally critical. Deep-learning models are often considered “black boxes,” which can hinder biological interpretation and regulatory approval. New techniques, such as saliency maps, feature attribution, and physics-informed neural networks, help identify the image features influencing predictions, linking computational results to underlying mechanisms. By improving transparency, these methods can build confidence among biologists and environmental engineers and support broader adoption of AI-enabled microfluidic systems.

### 5.2. Segmentation, Tracking, and Model Interpretability

Tracking in dense microenvironments remains computationally expensive and prone to error. Crowding, adhesion, and rapid directional switching challenge classical segmentation, particularly in low-SNR environments like 3D confocal channels or diffusion-only devices. While Transformer-based linkers (e.g., Trackastra) improve long-range associations, they remain dependent on high-quality segmentation masks. Future architectures must likely integrate instance segmentation with self-supervised representations that leverage motion cues to reduce dependence on perfect masking.

Beyond accuracy, interpretability is critical for biological acceptance. Deep-learning models are frequently criticized as “black boxes,” obscuring the link between image features and prediction. To counter this, techniques such as saliency maps and feature attribution are being employed to visualize decision-making processes. More promisingly, the field is moving toward hybrid modeling: combining data-driven predictions with mechanistic priors. Physics-Informed Neural Networks and Graph Neural Networks that incorporate advection-diffusion constraints allow us to align predictive outputs with biological hypotheses, such as receptor kinetics and quorum sensing.

### 5.3. Field-Deployable Systems and Real-Time Edge Computing

For microfluidics to impact real-world monitoring, systems must escape the laboratory. This requires the integration of microfluidics with portable imaging (e.g., smartphone microscopy) and low-power electronics [48,49]. However, current system integration is fragmented; variability in cartridge design and lighting limits model generalizability. To achieve robust field operation, we require standardized portable geometries, built-in calibration targets, and ruggedization against temperature fluctuations and vibration.

Simultaneously, the computational burden must be shifted to the edge. Real-time, division-aware tracking is currently too intensive for standard mobile processors. Techniques such as model distillation, quantization, and the use of lightweight backbones (e.g., MobileNet, YOLO-Nano) are essential to run analysis on embedded hardware accelerators. Frameworks like AI-CMCA demonstrate feasibility, but broader standardization is needed to enable low-latency toxicity readouts in resource-limited settings.

### 5.4. Integration with Synthetic Biology

The convergence of AI and microfluidics with synthetic biology presents a distinct frontier. Engineered microbes possessing tunable chemotaxis receptors and enhanced degradation pathways can now be screened under realistic environmental conditions [54,55]. AI-guided assays offer the ability to rapidly evaluate these variants, creating a feedback loop for the iterative design of bioremediation agents and biosensors. Realizing this potential requires harmonized protocols that can bridge the gap between biological fabrication and computational evaluation.

### 5.5. Ethical, Environmental, and Regulatory Considerations

The deployment of engineered microbes and autonomous diagnostic systems raises complex safety and ethical questions. From an ecological standpoint, rigorous biocontainment strategies and environmental risk assessments are necessary to prevent the unintended spread of engineered organisms. From a data perspective, regulatory frameworks increasingly demand explainability and provenance.

To gain regulatory trust for clinical or environmental decision-making, we must ensure reproducible pipelines by utilizing containerized environments and traceable model parameters. Furthermore, the physical footprint of these devices matters; sustainable materials and energy-efficient designs should be prioritized to minimize the ecological impact of disposable cartridges.

### 5.6. Future Outlook

The next decade suggests a convergence of microfluidics, embedded sensing, and quantitative biology. We anticipate the rise in autonomous, self-calibrating assays and AI-guided discovery platforms that can dynamically adapt to experimental data. Achieving these breakthroughs will require a coordinated effort to unify evaluation metrics, standardize datasets, and rigorously integrate physical laws into artificial intelligence models.

## 6. Conclusions

The integration of artificial intelligence and microfluidic technologies marks a transformative step in the study of cellular motility. By combining high-resolution imaging, automated analysis, and precise gradient control, these platforms enable quantitative and scalable chemotaxis assays that were previously unattainable. Beyond fundamental research, AI-enhanced microfluidic systems are now finding applications in environmental biotechnology, ranging from pollutant detection to predictive bioremediation, underscoring their growing societal relevance.

Looking ahead, the convergence of microfluidics, AI, and portable sensing is likely to reshape how motility assays are conducted across biology, medicine, and environmental science. Future capabilities will be driven by advances in microfabrication, such as 3D-printed devices, smart polymers, and integrated sensing electrodes, which will provide richer multimodal inputs ranging from mechanical deformation signatures to chemical reporter gradients. To fully leverage these hardware gains, the field must prioritize the standardization of datasets and the development of open-source analysis pipelines. This foundation will accelerate the adoption of advanced transformer-based tracking and physics-aware predictive models, ensuring that AI tools remain both interpretable and reproducible. As these technologies mature, rigorous field validation and the early resolution of ethical and ecological considerations will be critical for regulatory acceptance. Ultimately, the long-term vision is to develop autonomous, self-calibrating platforms capable of performing high-content analysis in clinical or field environments, serving as central tools for environmental monitoring, risk assessment, and sustainable engineering solutions.

## Figures and Tables

**Table 1:** AI methods for motility analysis in microfluidic assays (representative examples).

Method/Framework	Task & Context	Key Strengths & Limitations	Reported Performance	Ref.
TrackMate 7	General Tracking (Various assays)	User-friendly; DL-integrated Complex 3D needs plugins	Evaluated using Cell Tracking Challenge (CTC) metrics (such as tracking accuracy)	[32]
Cellpose 2.0	Segmentation (Phase-contrast channels)	Generalist; minimal tuning May need fine-tuning for rare phenotypes	AP at IoU = 0.5 (after 100–200 human-in-loop ROIs)	[33]
AI-CMCA (U-Net + MobileNet)	Fluid-front Detection (Capillary chips)	Edge-deployable; lightweight Task-specific (fluid only)	IoU > 0.99 Analysis up to 100× faster than manual	[34]
Trackastra (Transformer)	Linking/Tracking (Division-aware)	Learns mitosis; few hand-tuned params Requires substantial annotated training data	Evaluated using Cell Tracking Challenge (CTC) metrics (such as tracking accuracy)	[35]
Cell-TRACTR (Transformer)	End-to-End (Bacteria/Mammalian)	Joint segmentation + tracking High computational cost	Cell-HOTA ≈ 0.70	[36]
GNN Models	Collective Dynamics (Monolayer snapshots)	Predicts collective behavior Needs curated graph features	Area Under the Receiver Operating Characteristic Curve (AUROC) ≈ 0.90 for collective band-formation prediction	[37]
Transfer DL (Mask R-CNN)	Instance Detection (Droplet breakup)	Robust to clutter; data-efficient Requires domain adaptation	High AP Size Measurement Root Mean Square Error (RMSE) reduced by 30%	[38]

## Data Availability

No new data were created or analyzed in this study. Data sharing is not applicable to this article.
